# 培美曲塞/顺铂二线治疗晚期非小细胞肺癌：一例报告及文献复习

**DOI:** 10.3779/j.issn.1009-3419.2010.07.18

**Published:** 2010-07-20

**Authors:** 长林 赵, 红芹 孙, 扬 杨, 晓楠 代

**Affiliations:** 116021 大连，大连大学附属新华医院肿瘤科 Department of Medical Oncology, Xin Hua Hospital Affiliated to Dalian University, Dalian 116021, China

**Keywords:** 肺肿瘤, 晚期, 化学治疗, Lung neoplasms, Advanced, Chemotherapy

## Abstract

**背景与目的:**

结合文献复习，探讨二线治疗晚期非小细胞肺癌的价值。

**方法:**

对重组人血管内皮抑制素联合GC方案一线治疗获完全缓解（complete response, CR）后给予吉非替尼维持治疗，无进展生存期（progression free survival, PFS）10.2个月之后进展的1例转移性非小细胞肺腺癌患者，采用培美曲塞/顺铂二线治疗，随访观察患者PFS和生存时间。

**结果:**

培美曲塞/顺铂治疗5周期肺原发灶疗效为CR，骨转移灶稳定，PFS为6.6个月，至今已生存22个月，提高了患者的生活质量。

**结论:**

晚期非小细胞肺腺癌一线治疗/维持治疗后复发或转移，适时启动培美曲塞/顺铂二线治疗可延长患者生存期，提高生存质量。

## Abstract (en)

**Background and objective:**

The aim of this study is to integrate with literature review, and explore the value of treatment of advanced non-small cell lung cancer with second line.

**Methods:**

For the metastatic progressive non-small cell lung adenocarcinoma patient, the evaluation of efficacy for complete response (CR) with endostar combined GC, the sequential treatment with gefitinib, used pemetrexed combined DDP as the second line treatment, followed up and observed with the progression free survival (PFS) and survival time of patient.

**Results:**

Pemetrexed combined DDP in the treatment of 5 cycles, the evaluation of lung cancer efficacy for CR, bone metastasis was steady, PFS was 6.6 months, survival time is 22 months now, improved the quality of living life.

**Conclusion:**

For advanced non-small cell lung adenocarcinoma recurrence and metastasis, after the treatment of first line and maintenance therapy, selecting adequately pemetrexed combined DDP, as the second line treatment, can prolong the lifetime and improve the quality of life.

非小细胞肺癌（non-small cell lung cancer, NSCLC）患者在确诊时约70 %-80 %已处于晚期，其5年生存率约1.6%^[1]^。第三代化疗药物（紫杉醇、多西紫杉醇、吉西他滨、长春瑞滨）与铂类药物联合的一线化疗方案延长了晚期NSCLC患者的生存时间，1年生存率约25%-35%^[2]^，但总的生存时间仅延长2个月-4个月，大部分一线治疗有效的患者在较短时间内仍然会复发或转移，需进行二线治疗。我们对重组人血管内皮抑制素联合GC方案一线治疗完全缓解，后续吉非替尼维持治疗，无进展生存期（progression free survival, PFS）10.2个月后进展的1例转移性非小细胞肺腺癌患者，采用培美曲塞/顺铂二线治疗，延长了生存时间，提高了患者的生活质量，现结合文献复习报告如下。

## 1 临床资料

患者，女性，45岁。于2008年4月因刺激性咳嗽3个月，加重伴胸闷、气短1周入院。血CEA 86.02 ng/mL；B超示：右侧中等量胸腔积液，心包大量积液。肺MSCT平扫示：右肺中叶见大约11.1 cm×5.5 cm软组织肿块影，右肺中叶支气管呈截断改变，右肺上叶见磨玻璃密度影，左肺下叶见结节影，纵隔和隆突下淋巴结肿大。右侧胸腔和心包腔内见大量液体密度影。右侧胸穿置管引流胸水为渗出液，CEA 483.20 ng/mL。胸水涂片细胞学检查：找到腺癌细胞。心包穿刺置管引流出血性心包积液500 mL，CEA 497.18 ng/mL。纤维支气管镜检查镜下诊断：右肺中叶肺癌。活检病理诊断：肺中低分化腺癌。临床诊断：右肺中叶中心型NSCLC（pⅣ期T4N2M1），左肺转移，纵隔淋巴结转移，恶性胸腔积液、心包积液。体能状态评分（performance status, PS）采用美国东部肿瘤协作组（Eastern Cooperative Oncoloy Group, ECOG）PS（5分法）评分法。实体瘤（可测量病灶）近期疗效评价按RECIST（2000年）标准评价，完全缓解（complete response, CR）：全部肿瘤病灶消失≥4周；部分缓解（partial response, PR）：肿瘤病灶缩小30%以上≥4周；疾病稳定（stable disease, SD）：介于PR和PD之间；疾病进展（progressive disease, PD）：肿瘤病灶增大20%或出现新肿瘤病灶。毒性反应按NCI常见毒性分级标准（CTC 3.0版）评价，为0分-4分。本患者PS为4分。经全身最佳支持治疗PS达到2分，采用重组人血管内皮抑制素联合GC方案，伯尔定300 mg/m^2^，d1，吉西他滨（江苏豪森药业股份有限公司，批号：020105-050406）1 000 mg/m^2^，d1、d8；重组人血管内皮抑制素7.5 mg/m^2^，d1-d14，q 21 d×4。每治疗2周期，评价疗效和毒性反应。联合治疗4周期后，近期疗效评价为CR，以吉非替尼250 mg/d，维持治疗，后因经济原因中止维持治疗，PFS为10.2个月^[3]^。2009年6月再次出现干咳，胸闷、气急和胸骨区及右髋部疼痛。血CEA 430 ng/mL。肺MSCT平扫示：右肺中叶见大约5.5 cm×4.3 cm软组织肿块影，右侧胸腔和心包腔均示大量液体密度影。ECT和CT示骨转移。PS为4分。经右侧胸腔和心包腔穿刺置管引流等局部治疗和全身支持治疗后，PS达到1分，采用培美曲塞（江苏豪森药业股份有限公司，批号：H20051288）500 mg/m^2^，d1；顺铂75 mg/m^2^，q 21 d×5，给予叶酸和维生素B12、地塞米松预防不良反应。伊班膦酸6 mg/28天，治疗骨转移。治疗第2周期后，肺原发灶近期疗效为PR，右侧胸水2.0 cm，心包未见积液。治疗第4周期后，肺原发灶近期疗效为CR。培美曲塞/顺铂巩固治疗1周期。PFS为6.6个月，至今已生存22个月，提高了患者的生活质量。培美曲塞/顺铂二线治疗转移性NSCLC前后MSCT平扫结果（图 1）。

**1:**
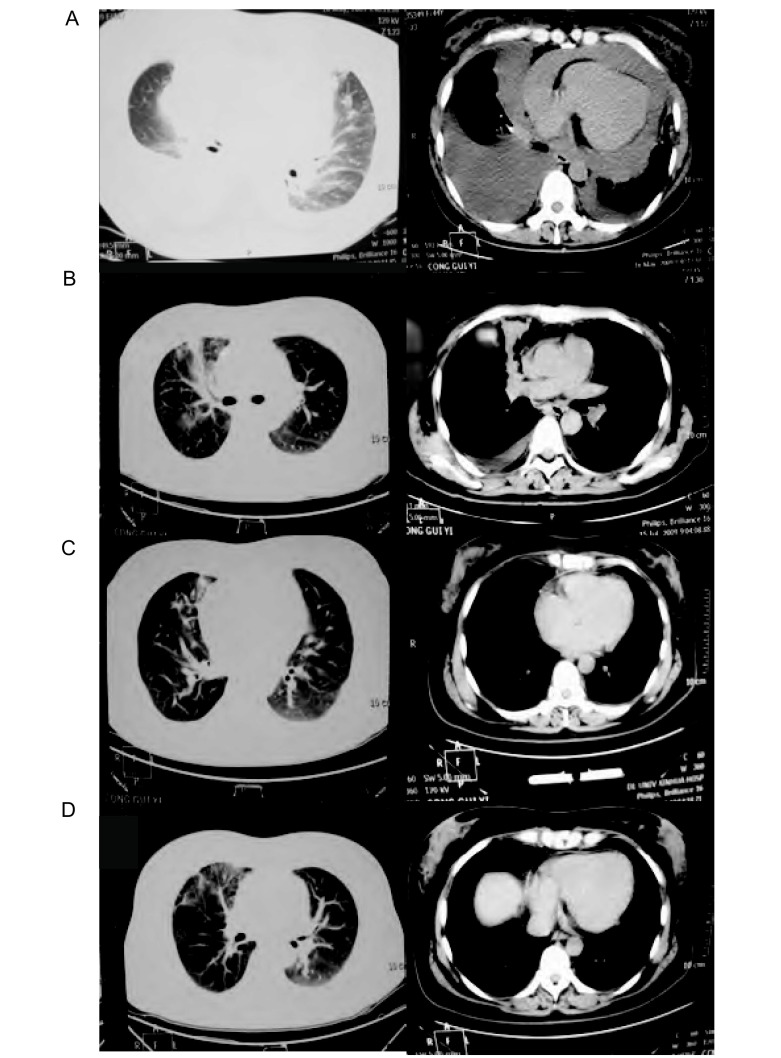
培美曲塞/顺铂二线治疗转移性NSCLC前后MSCT平扫结果。A：治疗前肺MSCT平扫（2009年5月16日）：右肺上叶见斑片状模糊影及中叶支气管狭窄，右肺上叶和中叶内侧段见三角形致密影, 5.5 cm×4.3 cm。纵隔内未见肿大淋巴结影，纵隔、心影略左移，双侧胸腔积液及心包大量积液；B：培美曲塞/顺铂治疗2周期，肺MSCT平扫示（2009年7月15日）：右肺上叶前段见不规则密度影，右肺中叶内侧段节段性不张，纵隔内未见异常肿大淋巴结影。右侧胸腔后壁见弧形水样密度影。近期疗效评价为PR；C：培美曲塞/顺铂治疗4周期，肺MSCT平扫示（2009年8月27日）：右肺见纤维索条影及斑片状密度增高影，纵隔内未见明确异常肿大影，近期疗效评价为CR；D：培美曲塞/顺铂巩固治疗1周期，肺MSCT平扫示（2009年11月5日）：右肺见斑片状密度增高影及纤维索条影，右肺中叶软组织肿块影消失，肺门支气管通畅。纵隔内未见异常肿大淋巴结影。近期评价为CR。 Results of MSCT before and after treatment of pemetrexed united DDP. A: Pre-treatment lung MSCT (May 16, 2009): right lobe showed around 5.5 cm×4.3 cm soft tissue mass shadow, thoracic and pericardial cavity, showed the liquid density; B: Pemetrexed combined DDP in the treatment of 2 cycles lung MSCT (July 15, 2009), lung MSCT scan showed: right superior lobe showed the irregular density, right middle lobe was segmental atelectasis. Right posterior wall of thoracic cavity showed curve imagine. Evaluation of short-term efficacy for PR; C: Pemetrexed combined DDP in the treatment of 4 cycles lung MSCT (August 27, 2009), lung MSCT scan showed: right lobe showed funiform and patchy shadowes of high density. Evaluation of short-term efficacy for CR; D: Pemetrexed combined DDP in the treatment of 5 cycles, right lobe showed patchy and funiform shadow, soft tissue mass shadow of right middle lobe disappeared, pulmonary hilum and bronchus opened. (November 5, 2009), lung MSCT scan evaluation for CR.

## 2 讨论

第三代化疗药物与含铂药物联合的一线治疗方案具有相似的客观缓解率（objetcive response rate, ORR）^[2]^。.对于明确有表皮生长因子受体（epidermal growth factor receptor, EGFR）活化突变或扩增，且无吸烟史的晚期NSCLC患者，酪氨酸激酶抑制剂（tyrosine kinase inhibitors, TKI）厄洛替尼单药或联合化疗可作为一线治疗的选择^[4]^。ECOG4599研究^[5]^结果表明，贝伐单抗与紫杉醇/卡铂联合作为一线治疗与单纯化疗相比，能显著改善晚期非鳞型NSCLC的PFS和ORR（29% *vs* 13%），成为治疗无出血史的晚期非鳞型NSCLC的标准一线治疗方案之一。JMDB研究^[6]^亚组分析显示，对于晚期非鳞型NSCLC患者，培美曲塞/顺铂组在总生存期（overall survival, OS）方面优于吉西他滨/顺铂组，且毒性更低。FLEX研究^[7]^表明，一线标准化疗联合西妥昔单抗，可使所有组织学亚型患者的生存期显著延长。西妥昔单抗联合长春瑞滨/顺铂的方案较单纯化疗可以显著改善ORR（36% *vs* 29%），使含铂两药联合一线治疗的金标准受到挑战。重组人血管内皮抑制素联合长春瑞滨/顺铂与单纯化疗相比，PFS的临床获益率均优于单药化疗^[8]^。2009年《美国国家综合癌症网（National Comprehensive Cancer Network, NCCN）非小细胞肺癌指南》中规定，对于复发或转移性NSCLC患者，ECOG为0分-1分者，可给予贝伐单抗联合化疗、培美曲塞/顺铂或西妥昔单抗/长春瑞滨/顺铂治疗；ECOG为2分者，可给予西妥昔单抗/长春瑞滨/顺铂或单纯化疗；ECOG为3分-4分者，给予最佳支持治疗；对有*KRAS*突变者应考虑厄洛替尼以外的治疗^[9-11]^。本例为中年女性患者，无吸烟史，于2008年确诊为Ⅳ期腺癌型NSCLC，经最佳支持治疗ECOG达到2分后，一线治疗采用重组人血管内皮抑制素联合GC方案4周期疗效达CR，后续吉非替尼维持治疗，PFS达10.2个月，提示重组人血管内皮抑制素联合GC方案治疗有效后，给予吉非替尼维持治疗可以延长女性非吸烟Ⅳ期肺腺癌患者的PFS。对于一线治疗失败的晚期或转移性NSCLC，如果ECOG为0分-1分者，二线治疗可选择的化疗药物有培美曲塞、多西紫杉醇；TKI有吉非替尼和厄洛替尼；增加了白蛋白结合型紫杉醇、西妥昔单抗^[9-11]^。Scagliotti等^[12]^研究结果显示，在一线治疗失败的晚期非鳞型NSCLC的二线治疗中，培美曲塞中位OS优于多西紫杉醇，且毒性更低，认为培美曲塞可作为标准二线治疗方案。Reck等^[13]^在INTEREST研究中比较了晚期NSCLC二线治疗方案，证明了吉非替尼与多西紫杉醇在ORR和OS方面无差异，吉非替尼在毒副反应和生活质量改善方面，均优于多西紫杉醇，并可适用于各种类型的NSCLC。Herbst等^[14]^在晚期NSCLC二线治疗的ZODIAC试验中，将凡德他尼联合多西紫杉醇与多西紫杉醇联合安慰剂相比，前者可以延长PFS、ORR（17% *vs* 10%）。凡德他尼可单药或联合多西紫杉醇作为二线治疗。尽管这些研究结果令人振奋，但作为二线治疗的药物和方案在OS方面的孰优孰劣尚无定论。本例患者二线治疗选择了培美曲塞/顺铂治疗4周期肺原发灶近期疗效为CR，巩固治疗1周。PFS为6.6个月，至今已生存22个月。因此，研究认为当患者出现肿瘤相关症状或肿瘤进展时启动二线治疗是合理的决策，二线治疗药物和方案的选择应该根据患者的ECOG评分、组织类型、基因检测结果和经济状况等不同个体，并尊重患者的意愿，将合适的（right treatment）二线治疗药物和方案，适时（right time）适度地用于合适的患者（right patient），对疗效的最大化至关重要。
